# Application of Three-Dimensional Pseudocontinuous Arterial Spin Labeling Perfusion Imaging in the Brains of Children With Autism

**DOI:** 10.3389/fneur.2022.851430

**Published:** 2022-02-23

**Authors:** Shilong Tang, Xianfan Liu, Qiying Ran, Lisha Nie, Lan Wu, Zhengxia Pan, Ling He

**Affiliations:** ^1^Department of Radiology Children's Hospital of Chongqing Medical University, National Clinical Research Center for Child Health and Disorders, Ministry of Education Key Laboratory of Child Development and Disorders, Chongqing Key Laboratory of Pediatrics, Chongqing, China; ^2^GE Healthcare, MR Research China, Beijing, China; ^3^Department of Cardiovascular and Thoracic Surgery, Children's Hospital of Chongqing Medical University, Chongqing, China

**Keywords:** brain, MRI, children, autism, three-dimensional arterial spin labeling

## Abstract

**Objective:**

To explore the application of three-dimensional pseudocontinuous arterial spin labeling (3D-PCASL) perfusion imaging in the brains of children with autism and to understand the characteristics of cerebral blood perfusion in children with autism.

**Methods:**

A total of 320 children with autism (160 men and 160 women) aged between 2 and 18 years and 320 age- and sex-matched healthy children participated in the study. All children were scanned by 3.0 T magnetic resonance axial T1 fluid-attenuated inversion recovery (FLAIR), T2 FLAIR, 3D-T1, and 3D-PCASL sequences. After postprocessing, cerebral blood flow (CBF) values in each brain region of children with autism and healthy children at the same age were compared and analyzed. Furthermore, CBF characteristics in each brain region of autistic children at various ages were determined.

**Results:**

The CBF values of the frontal lobe, hippocampus, temporal lobe, and caudate nucleus of children with autism are lower than those of healthy children (*P* < 0.05). Additionally, as the ages of children with autism increase, the number of brain regions with decreased CBF values gradually increases. A receiver operating characteristic (ROC) analysis results show that the CBF values of the frontal lobe, hippocampus, temporal lobe, and caudate nucleus can distinguish children with autism [area under the ROC curve (AUC) > 0.05, *P* < 0.05].

**Conclusion:**

The 3D-PCASL shows lower brain CBF values in children with autism.

**Clinical Trial Registration:**

www.ClinicalTrials.gov, identifier: ChiCTR2000034356.

## Key Points

- Three-dimensional pseudocontinuous arterial spin labeling shows lower brain CBF values in children with autism.
- The temporal lobe, frontal lobe, hippocampus, and caudate nucleus can distinguish children with autism [area under the ROC curve (AUC) > 0.5, *P* < 0.05].
- The temporal lobe, frontal lobe, hippocampus, and caudate nucleus regions may be the first areas to show cerebral blood flow changes in autistic children.

## Introduction

Autism spectrum disorder (ASD) refers to a group of developmental disorders that start in early childhood, with high incidence and unclear causes (1). ASD is a multifactorial disease related to genetics, neurodevelopment, social psychology, etc. (2, 3). Some pathogenic factors have not been clearly identified (4, 5). For example, studies have found that some brain regions of children with autism have abnormal anatomical structures or abnormal brain function activities (6, 7), but whether cerebral blood perfusion in abnormal brain regions is also abnormal is unknown.

In previous studies, some researchers have used single-photon emission computed tomography (SPECT) to determine cerebral blood perfusion in children with ASD (8, 9). SPECT is an instrument that uses radioisotopes as tracers to obtain blood flow values in various regions of children's brains, which is harmful to the human body, and children are at a high risk of adverse effects due to γ-ray (radioactive isotope decay produces γ- ray) radiation exposure (10, 11). Therefore, identifying a technology with no impact on children's bodies to determine their brain blood flow is a study hotspot for medical researchers.

Three-dimensional pseudocontinuous arterial spin labeling (3D-PCASL) is a technique for image perfusion that uses continuous labeling (12, 13). This technique is non-invasive and can be used to perform functional imaging and evaluation of the whole brain repeatedly without the need for contrast agent injection. It has been widely used in clinical practice (14, 15). At present, more reports are available on the application of 3D-PCASL technology to adult brain examinations (16), while fewer reports are available on its application to pediatric brain examinations, with an especially low number of reports on its application to head examinations in children with autism (17, 18).

In this study, 3D-PCASL cerebral blood perfusion imaging was used to obtain the blood perfusion value of each brain region of the children's brains. The blood perfusion values of various brain regions of children with autism and healthy children aged 2–18 years were compared and analyzed to determine differences in the blood perfusion value of each brain area between children with autism and healthy children such that the brain blood perfusion characteristics of children with autism can be accurately identified as soon as possible, children with autism can receive a timely diagnosis and reasonable treatment, the severity of the illness can be improved, and the burden on the family and society can be reduced.

## Materials and Methods

### Ethics Statement

The study protocol was approved by the Human Ethics Committee of the Children's Hospital of Chongqing Medical University (*No. 2018-47*). Written informed consent was obtained from the parents or guardians of all the children before the examinations.

### Study Participants

Study group: A total of 431 children with autism aged 2 to 18 years were selected, from June 2018 to March 2021, and 320 children (men 160, women 160) were included in the study. Control group: A total of 401 healthy children aged 2 to 18 years were selected from February 2019 to April 2021, and 320 children (men 160, women 160) were included in the study. Both groups of children were divided into eight subgroups with 40 in each subgroup according to age (2, 3, 4, 5, 6–8, 9–10, 11–14, 15–18 years old). Children who were excluded from the study had abnormal brain lesions or did not cooperate with sedation, resulting in breathing artifacts on imaging (Table 1).

**Table 1:** Patient information.

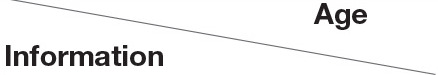		**2 years old**	**3 years old**	**4 years old**	**5 years old**	**6–8 years old**	**9–10 years old**	**11–14 years old**	**15–18 years old**
Male to female ratio	Healthy children	20:20	20:20	20:20	20:20	20:20	20:20	20:20	20:20
	Autistic children	20:20	20:20	20:20	20:20	20:20	20:20	20:20	20:20
	*X*^2^ value	0.000	0.000	0.000	0.000	0.000	0.000	0.000	0.000
	*P*-value	1.000	1.000	1.000	1.000	1.000	1.000	1.000	1.000
Age	Healthy children	2.4 ± 0.6	3.2 ± 0.3	4.2 ± 0.7	5.3 ± 0.3	7.1 ± 1.0	8.8 ± 1.2	12.5 ± 1.9	15.9 ± 2.1
	Autistic children	2.3 ± 0.5	3.4 ± 0.5	4.3 ± 0.4	5.3 ± 0.4	7.2 ± 1.3	8.6 ± 1.3	12.4 ± 1.8	15.6 ± 1.9
	T value	0.874	3.635	7.982	2.877	3.653	2.879	1.984	4.567
	*P*-value	0.763	0.074	0.238	0.229	0.083	0.764	0.432	0.095
BMI	Healthy children	15.9 ± 1.8	15.4 ± 2.3	15.1 ± 1.6	14.9 ± 2.9	15.2 ± 3.2	15.8 ± 4.7	18.6 ± 4.7	20.1 ± 5.6
	Autistic children	15.7 ± 1.6	15.6 ± 2.1	15.0 ± 1.3	14.7 ± 2.1	15.1 ± 2.9	15.6 ± 5.6	18.4 ± 5.1	20.0 ± 6.9
	T value	7.653	4.786	3.254	5.124	6.259	1.887	3.239	4.665
	*P*-value	0.065	0.128	0.709	0.237	0.982	0.105	0.876	0.237
Weight	Healthy children	16.6 ± 1.5	17.4 ± 1.2	16.7 ± 1.5	17.8 ± 1.3	16.3 ± 1.6	17.1 ± 1.0	18.67 ± 1.36	21.2 ± 1.5
	Autistic children	16.2 ± 1.3	17.4 ± 1.3	16.5 ± 1.2	17.2 ± 1.2	16.3 ± 1.4	17.1 ± 1.1	18.5 ± 1.2	21.3 ± 1.6
	T value	3.876	2.983	1.668	7.982	2.685	3.298	4.652	1.993
	*P*-value	0.087	0.982	0.073	0.134	0.076	0.981	0.091	0.553
Sedation needed	Healthy children	40	40	36	22	0	0	0	0
	Autistic children	40	40	40	38	21	3	3	0

The inclusion criteria for children in the control group: a body mass index of 15–18 kg/m^2^, right-handedness, no neurological disease, no other organ-related diseases, no other diseases that may affect brain function and structure, and no abnormalities on routine head MRI.

The inclusion criteria for the children in the study group: a body mass index of 15–18 kg/m^2^ and right-handedness. The children were required to meet the diagnostic criteria of the Diagnostic and Statistical Manual of Mental Disorders (DSM-V) for autism (19), with no neurological disease, no other organ-related diseases, no other diseases that may affect brain function and structure, no history of medical treatment, and no abnormalities on routine head MRI; the children with ASD in the study group were diagnosed at the outpatient clinic of a physician with an associate senior title or above in the Children's Developmental Behavior and Child Health Department and the Department of Psychology of Children's Hospital of Chongqing Medical University. Children with ASD included in the study had to meet the diagnostic criteria for autism in the fifth edition of the *Diagnostic and Statistical Manual of Mental Disorders* and have a Childhood Autism Rating Scale (CARS) score ≥30.

All children who did not cooperate with the examination were examined in the sedation center of our hospital after sedation. The sedation method was as follows: dexmedetomidine 3 μg/kg intranasally + chloral hydrate 40 mg/kg orally. If the sedation depth was not sufficient after 20 min, dexmedetomidine 1 μg/kg was administered intranasally again, and if necessary, chloral hydrate 20 mg/kg was administered orally.

### Imaging Data Collection and Postprocessing

In this study, a GE 3.0 T magnetic resonance (Discovery MR750; GE Medical Systems, Milwaukee, WI, USA) scanner with an eight-channel head-neck joint coil was used. Children who did not cooperate with the examination were sent to the sedation center for sedation and axial T1 FLAIR, T2 FLAIR, T2 weighted imaging (WI), 3D-T1, and 3D-PCASL sequence scans after deep sleep were achieved. 3D-PCASL: repetition time (TR): 4,628 ms, field of view (FOV): 25 cm, time of echo (TE): 10.4 ms, number of excitations (NEX): three times, slice thickness: 4.2 mm, 32 slices, post labeling delay (PLD): 1,525 ms, and scanning time: 4 min 29 s. 3D-T1: TR: 450 ms, FOV: 25 cm, TE: 3.1 ms, NEX: 1 time, slice thickness: 1 mm, 152 slices, and scanning time: 3 min 43 s. The range of all sequence scanning was from skull top to skull base, including the whole brain.

#### Data Analysis

**Figure 1:**
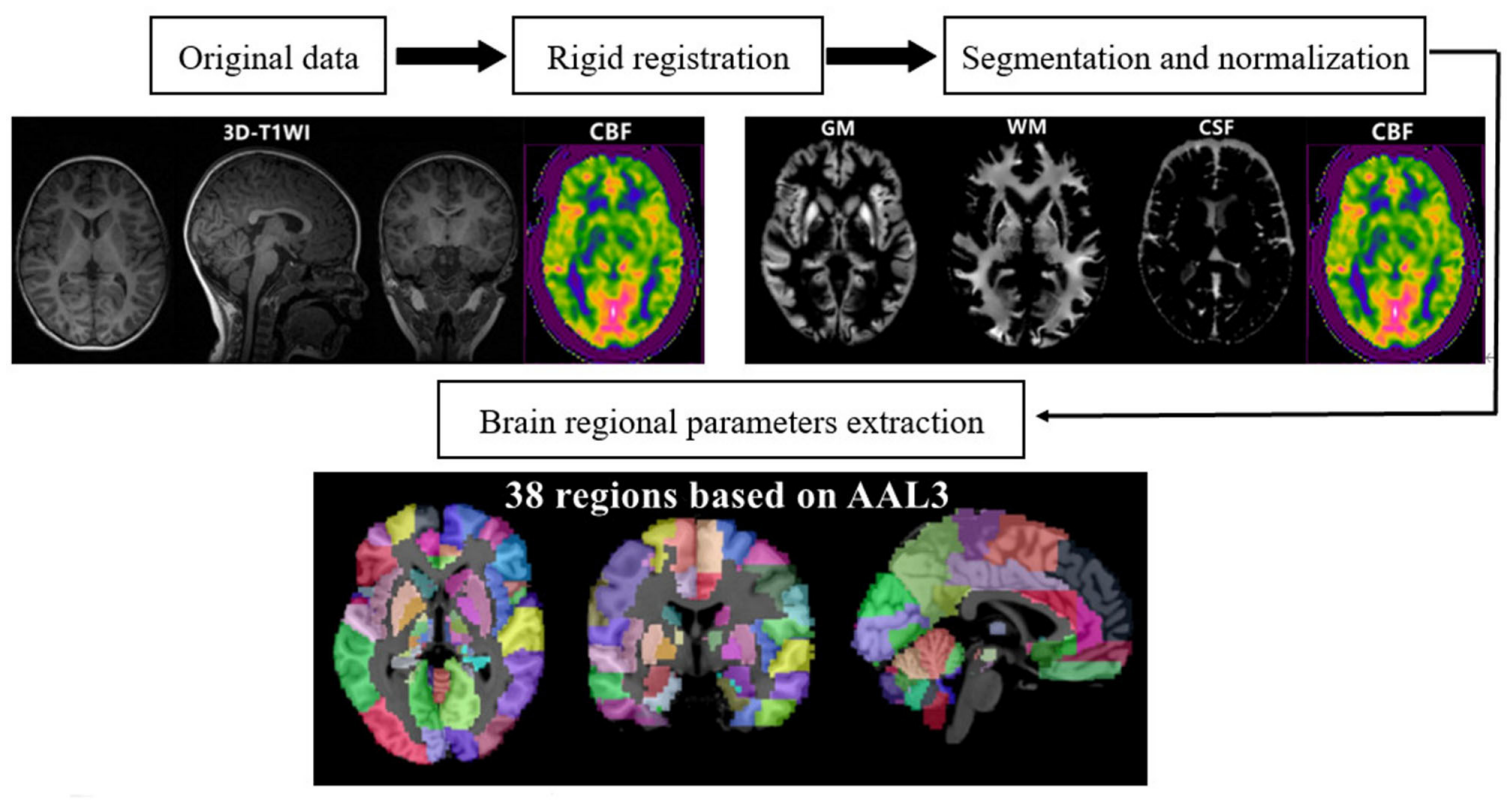
Schematic diagram of brain volume and CBF parameter extraction.

**Figure 2:**
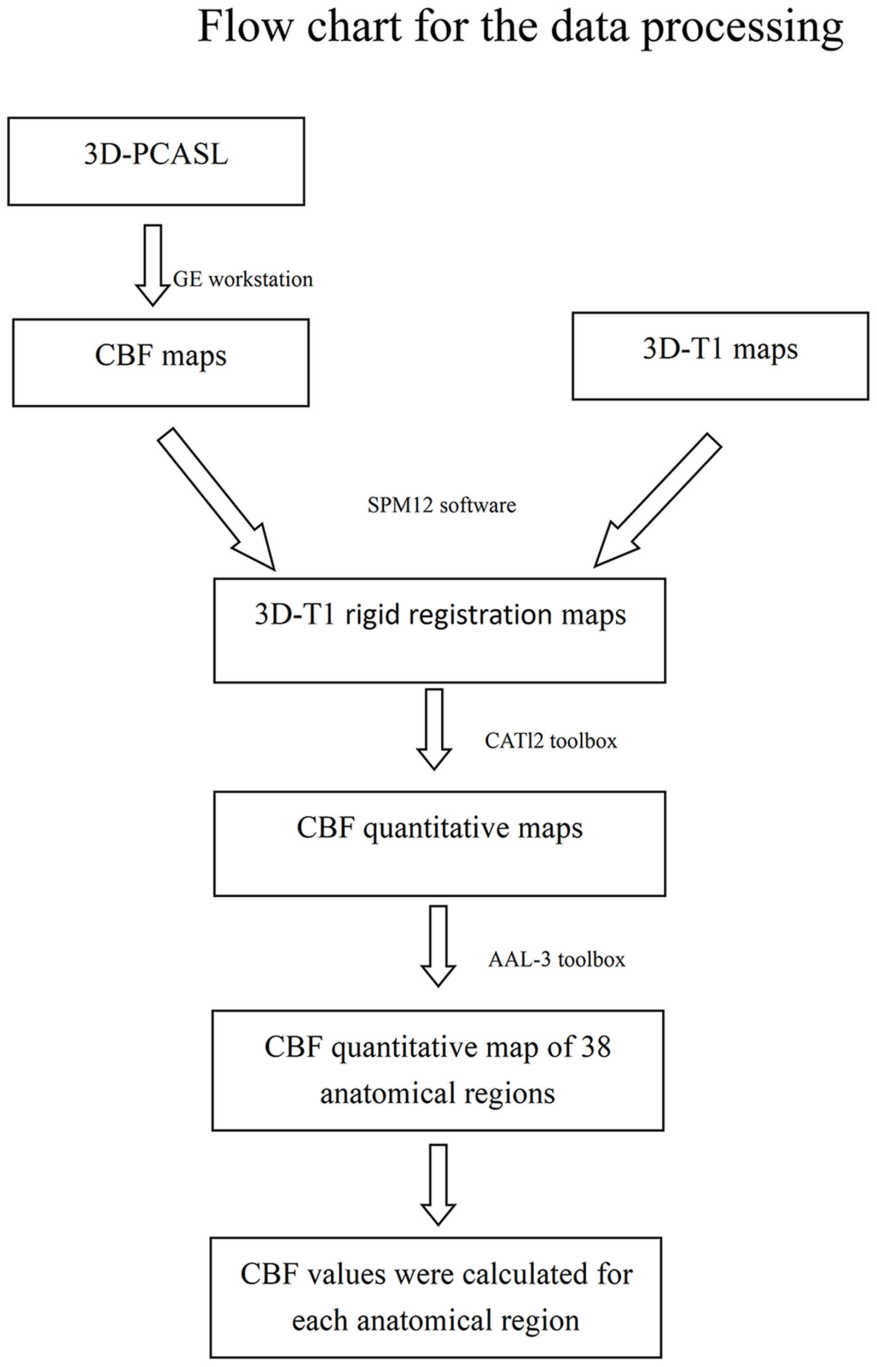
Flow chart for the data processing.

To calculate the quantitative parameters, including the gray matter volume (GMV), white matter volume (WMV), and CBF values of different brain regions, we followed an atlas-based image processing approach. CBF maps were further calculated from the 3D-PCASL data using an AW4.6 GE Workstation. Second, we performed rigid registration between CBF maps and 3D-T1WI data using SPM12 software (http://www.fil.ion.ucl.ac.uk/spm/) based on MATLAB (MathWorks, Natick, MA, USA). Third, 3D-T1WI images were segmented and nonlinearly normalized into MNI space using the CAT12 toolbox (http://www.neuro.uni-jena.de/cat/) implemented in SPM12 to obtain tissue probability maps and normalized CBF quantitative maps. Finally, the brain was parcellated into 38 anatomical regions based on the automated anatomical labeling atlas (AAL-3) for quantitative parameter extraction. Brain regional volumes and CBF can be extracted by averaging the values from voxels in specific brain regions. The estimated global GMV and WMV were further normalized by correction of the intracranial volume (Figures 1, 2).

### Statistical Analysis

In this study, SPSS 25.0 statistical software was used, and the measurement data were expressed as the mean and standard deviation. The 3D-PCASL perfusion CBF value of the same brain region in children of the same age in the study group and the control group was compared by two-independent samples *t*-tests (*P* < 0.05 indicates that a difference was statistically significant); the volume of the same brain region in children of the same age in the study group and the control group was compared by two-independent samples *t*-tests (*P* < 0.05 indicates that a difference was statistically significant).

A receiver operating characteristic (ROC) curve was used to evaluate the diagnostic value of the CBF value for the diagnosis of childhood autism. An area under the ROC curve (AUC) > 0.5 with statistical significance was considered indicative of diagnostic value. A value closer to one corresponded to a higher diagnostic value.

## Results

### Comparison of CBF Values in the Same Age Group and the Same Brain Region

**Figure 3:**
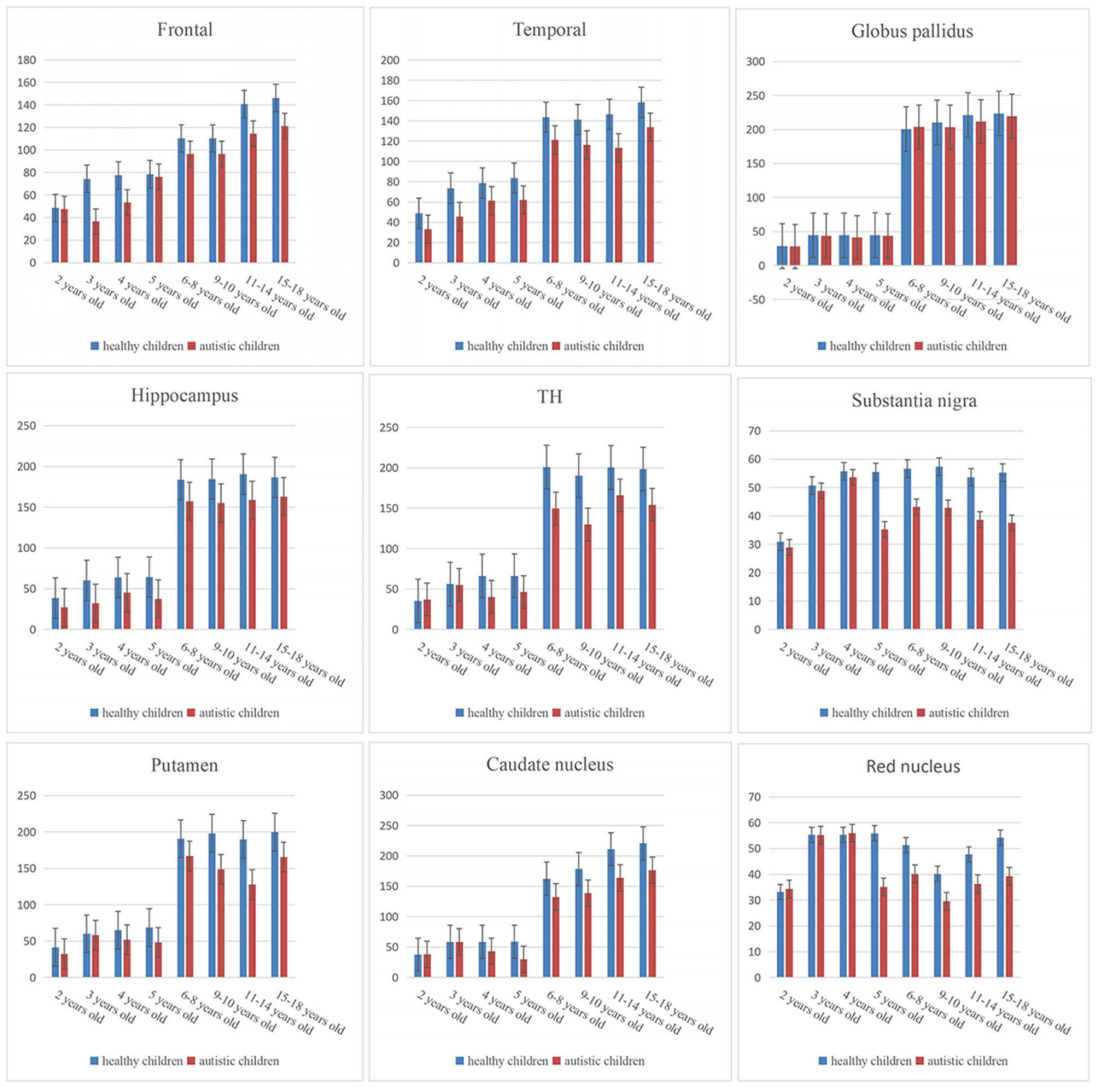
Bar chart of CBF values of brain regions in children.

**Figure 4:**
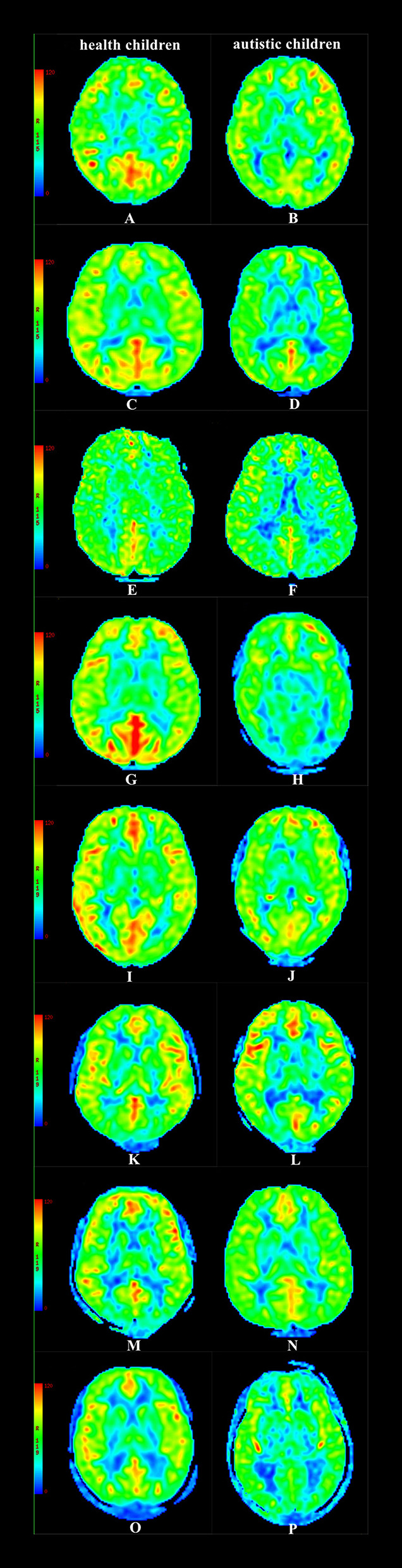
CBF maps **(A)** Male, 2.3 y, healthy children. **(B)** Female, 2.4 y, autistic children. **(C)** Male, 3.3 y, healthy children. **(D)** Male, 3.6 y, autistic children. **(E)** Female, 4.5 y, healthy children. **(F)** Male, 4.4 y, autistic children. **(G)** Male, 5.6 y, healthy children. **(H)** Male, 5.4 y, autistic children. **(I)** Female, 6.9 y, healthy children. **(J)** Male, 6.7 y, autistic children. **(K)** Male, 9.7 y, healthy children. **(L)** Male, 9.9 y, autistic children. **(M)** Male, 12.7 y, healthy children. **(N)** Famle, 12.8 y, autistic children. **(O)** Male, 16.8 y, healthy children. **(P)** Male, 16.6 y, autistic children.

In the group with 2-year-old children, the CBF values of the temporal lobe, hippocampus, and putamen in children with autism were lower than those in healthy children (*P* < 0.05); in the group containing 3-year-old children, the CBF values of the frontal lobe, temporal lobe, hippocampus, and putamen in children with autism were lower than those in healthy children (*P* < 0.05); in the group with 4-year-old children, the CBF values of the temporal lobe, frontal lobe, thalamus, hippocampus, putamen, and caudate nucleus in children with autism were lower than those in healthy children (*P* < 0.05); in the group containing 5-year-old children, the CBF values of the temporal lobe, thalamus, hippocampus, putamen, caudate nucleus, substantia nigra, and red nucleus in children with autism were lower than those in healthy children (*P* < 0.05); and in the group with 6–18-year-old subjects, the CBF values of the frontal lobe, temporal lobe, thalamus, hippocampus, caudate nucleus, substantia nigra, and red nucleus in children with autism were lower than those in healthy children (*P* < 0.05) (Table 2, Figures 3, 4).

**Table 2:** CBF values of brain regions in children [CBF (ml/100 g·min), x ± s, *n* = 40].

**Brain regions**	**CBF (ml/100 g·min)**		***P*** **-value**	**Brain regions**	**CBF (ml/100 g·min)**		***P*** **-value**
	**Healthy children**	**Autistic children**			**Healthy children**	**Autistic children**	
**2 years old**				**3 years old**			
Frontal	48.6 ± 8.1	47.5 ± 6.2	NS	Frontal	74.5 ± 7.1	36.5 ± 6.3	**<0.001**
Temporal	48.9 ± 7.7	33.2 ± 5.9	**<0.001**	Temporal	73.6 ± 6.9	45.6 ± 7.5	**<0.001**
Hippocampus	38.7 ± 6.2	27.1 ± 4.9	**<0.001**	Hippocampus	60.2 ± 8.5	32.1 ± 5.6	**<0.001**
TH	35.2 ± 4.5	37.1 ± 5.0	NS	TH	56.1 ± 6.1	55.2 ± 8.1	NS
GP	28.7 ± 5.1	28.1 ± 6.2	NS	GP	44.5 ± 5.1	43.7 ± 6.1	NS
SN	30.9 ± 7.9	28.9 ± 6.9	NS	SN	50.7 ± 7.9	48.8 ± 3.6	NS
Putamen	41.7 ± 6.1	32.6 ± 5.1	**<0.001**	Putamen	60.2 ± 9.1	58.4 ± 8.1	**<0.001**
RN	33.2 ± 6.2	34.3 ± 5.1	NS	RN	55.3 ± 6.1	55.2 ± 6.3	NS
CN	37.7 ± 6.3	38.0 ± 5.7	NS	CN	58.6 ± 7.1	58.7 ± 8.5	NS
**4 years old**				**5 years old**			
Frontal	77.5 ± 7.5	53.4 ± 7.6	**<0.001**	Frontal	78.5 ± 7.5	76.2 ± 6.5	NS
Temporal	78.6 ± 6.4	61.2 ± 6.1	**<0.001**	Temporal	83.6 ± 6.5	62.1 ± 7.8	**<0.001**
Hippocampus	64.1 ± 8.2	45.3 ± 9.2	**<0.001**	Hippocampus	64.5 ± 8.2	37.6 ± 9.1	**<0.001**
TH	66.1 ± 6.7	40.1 ± 7.4	**<0.001**	TH	66.3 ± 6.7	46.2 ± 9.6	**<0.001**
GP	44.5 ± 5.3	41.2 ± 6.9	NS	GP	44.8 ± 5.3	43.7 ± 6.9	NS
SN	55.7 ± 7.6	53.6 ± 8.2	NS	SN	55.5 ± 7.6	35.2 ± 8.1	**<0.001**
Putamen	65.2 ± 9.4	52.1 ± 4.8	**<0.001**	Putamen	68.7 ± 9.4	48.2 ± 7.6	**<0.001**
RN	55.3 ± 6.7	56.0 ± 8.4	NS	RN	55.9 ± 6.7	35.1 ± 6.5	**<0.001**
CN	58.6 ± 7.9	43.1 ± 9.1	**<0.001**	CN	58.8 ± 7.9	30.2 ± 9.1	**<0.001**
**6–8 years old**				**9–10 years old**			
Frontal	110.3 ± 0.11.6	96.5 ± 18.6	**<0.001**	Frontal	140.6 ± 37.6	122.3 ± 26.5	**<0.001**
Temporal	143.6 ± 37.6	121.2 ± 27.6	**<0.001**	Temporal	141.3 ± 41.5	116.4 ± 23.6	**<0.001**
Hippocampus	183.9 ± 28.9	157.4 ± 33.2	**<0.001**	Hippocampus	184.4 ± 38.6	155.2 ± 34.5	**<0.001**
TH	200.8 ± 36.2	149.5 ± 40.2	**<0.001**	TH	190.2 ± 32.5	129.8 ± 32.1	**<0.001**
GP	200.4 ± 41.2	203.7 ± 37.4	NS	GP	210.3 ± 22.9	203.4 ± 43.1	NS
SN	56.7 ± 8.5	43.2 ± 9.6	**<0.001**	SN	57.4 ± 11.5	42.8 ± 8.2	**<0.001**
Putamen	190.9 ± 21.8	166.9 ± 36.6	**<0.001**	Putamen	198.3 ± 40.5	148.9 ± 36.5	**<0.001**
RN	51.3 ± 9.6	40.2 ± 8.9	**<0.001**	RN	40.2 ± 7.6	29.6 ± 8.2	**<0.001**
CN	162.4 ± 32.4	132.5 ± 27.6	**<0.001**	CN	178.6 ± 31.4	138.6 ± 29.5	**<0.001**
**11–14 years old**				**15–18 years old**			
Frontal	140.78 ± 35.6	114.62 ± 30.5	**<0.001**	Frontal	146.23 ± 33.4	121.34 ± 37.4	**<0.001**
Temporal	146.52 ± 26.3	113.43 ± 27.6	**<0.001**	Temporal	158.27 ± 29.8	133.65 ± 33.6	**<0.001**
Hippocampus	190.47 ± 37.7	158.66 ± 28.9	**<0.001**	Hippocampus	186.63 ± 37.4	163.11 ± 41.2	**<0.001**
TH	200.32 ± 41.5	165.79 ± 40.5	**<0.001**	TH	198.54 ± 38.5	154.23 ± 44.7	**<0.001**
GP	220.98 ± 22.3	211.65 ± 43.2	NS	GP	223.68 ± 42.6	219.63 ± 47.8	NS
SN	53.65 ± 23.6	38.69 ± 13.6	**<0.001**	SN	55.29 ± 15.7	37.56 ± 12.3	**<0.001**
Putamen	189.74 ± 26.9	127.58 ± 37.8	**<0.001**	Putamen	199.87 ± 40.5	165.78 ± 33.7	**<0.001**
RN	47.71 ± 15.2	36.33 ± 13.9	**<0.001**	RN	54.21 ± 21.4	39.24 ± 12.5	**<0.001**
CN	210.87 ± 43.2	164.17 ± 37.6	**<0.001**	CN	220.65 ± 55.5	176.56 ± 55.6	**<0.001**

*TH, Thalamus; GP, Globus pallidus; SN, Substantia nigra; RN, Red nucleus; CN, Caudate nucleus. P < 0.05 indicates that a difference was statistically significant*.

### Volume Comparison of the Same Age Group and the Same Brain Region

In the group with 2–5-year-old children, no statistically significant difference in the volume of the same brain region was found between children with autism and healthy children in the same age group; in the group containing 6- to 8-year-olds, the volumes of the frontal lobe, temporal lobe, hippocampus, and putamen in healthy children were smaller than those in children with autism (*P* < 0.05); in the group with 9- to 10-year-old children, the volumes of the temporal lobe, frontal lobe, thalamus, hippocampus, putamen, and caudate nucleus in healthy children were smaller than those in children with autism (*P* < 0.05); in the group with 11-to 18-year-old subjects, the volumes of the frontal lobe, temporal lobe, thalamus, hippocampus, putamen, caudate nucleus, substantia nigra, and red nucleus in healthy children were smaller than those in children with autism (*P* < 0.05) (Table 3).

**Table 3:** Volume values of brain regions in children [volume (cm^3^), x ± s, *n* = 40].

**Brain regions**	**Volume (cm** ^**3**^ **)**		**T value**	***P*** **-value**	**Brain regions**	**Volume (cm** ^**3**^ **)**		**T value**	***P*** **-value**
	**Control group**	**Study group**				**Control group**	**Study group**		
**≥2–≤3 years old**					**≥3–≤4 years old**				
Frontal	88.60 ± 16.13	87.56 ± 11.53	18.876	0.238	Frontal	104.57 ± 16.57	107.13 ± 12.69	21.563	0.563
Temporal	87.85 ± 13.81	85.47 ± 9.66	16.125	0.093	Temporal	100.24 ± 15.21	98.57 ± 17.34	16.873	0.238
Hippocampus	7.75 ± 2.12	7.24 ± 2.31	36.421	0.982	Hippocampus	7.39 ± 2.87	7.22 ± 1.69	3.894	0.076
TH	8.15 ± 2.01	8.29 ± 2.34	5.661	0.874	TH	8.15 ± 2.24	8.26 ± 1.83	4.652	0.237
GP	1.56 ± 0.89	1.47 ± 1.01	1.134	0.094	GP	1.56 ± 0.69	1.45 ± 0.33	1.097	0.125
SN	0.13 ± 0.03	0.12 ± 0.2	0.457	0.067	SN	0.13 ± 0.07	0.11 ± 0.04	0.873	0.802
Putamen	10.65 ± 3.23	10.18 ± 2.83	2.873	0.137	Putamen	10.75 ± 3.01	10.11 ± 2.98	7.661	0.236
RN	0.0045 ± 0.0003	0.0042 ± 0.0002	0.451	0.087	RN	0.0046 ± 0.0002	0.0043 ± 0.0002	0.874	0.076
CN	6.24 ± 1.27	6.16 ± 1.31	3.284	0.076	CN	6.51 ± 1.89	6.37 ± 1.56	3.287	0.087
Whole brain volume	1266.89 ± 53.63	1254.76 ± 87.69	15.869	0.095	Whole brain volume	1287.64 ± 93.42	1301.64 ± 89.87	24.876	0.325
**≥4–≤5 years old**					**≥5–≤6 years old**				
Frontal	100.58 ± 14.25	98.24 ± 16.87	13.897	0.671	Frontal	98.64 ± 8.97	99.67 ± 7.63	17.675	0.993
Temporal	109.23 ± 23.58	100.59 ± 25.13	16.657	0.084	Temporal	105.57 ± 21.83	101.62 ± 18.92	24.773	0.097
Hippocampus	7.56 ± 3.24	7.31 ± 1.64	8.378	0.981	Hippocampus	7.54 ± 2.16	7.45 ± 1.88	1.873	0.084
TH	9.29 ± 2.35	8.75 ± 2.62	8.391	0.265	TH	9.22 ± 1.96	9.13 ± 1.62	3.452	0.075
GP	1.6 ± 0.34	1.47 ± 0.38	3.124	0.871	GP	1.57 ± 0.41	1.61 ± 0.37	0.873	0.085
SN	0.14 ± 0.05	0.13 ± 0.05	0.675	0.061	SN	0.15 ± 0.03	0.16 ± 0.04	0.321	0.114
Putamen	11.13 ± 3.21	10.99 ± 3.14	3.872	0.105	Putamen	11.26 ± 3.42	10.89 ± 3.65	6.762	0.287
RN	0.0046 ± 0.003	0.0041 ± 0.0002	0.134	0.087	RN	0.0047 ± 0.001	0.0051 ± 0.002	0.875	0.271
CN	7.15 ± 1.53	6.69 ± 1.03	4.235	0.109	CN	7.42 ± 1.36	7.33 ± 1.64	2.654	0.139
Whole brain volume	1354.69 ± 58.65	1296.55 ± 88.63	15.632	0.983	Whole brain volume	1399.87 ± 87.83	1368.59 ± 89.88	18.994	0.882
**≥6–≤8 years old**					**≥8–≤10 years old**				
Frontal	163.23 ± 21.45	177.57 ± 32.56	8.367	<0.001	Frontal	162.57 ± 41.54	178.46 ± 37.62	0.993	<0.001
Temporal	144.45 ± 32.65	156.66 ± 29.87	17.652	<0.001	Temporal	149.25 ± 35.63	159.97 ± 37.65	0.874	<0.001
Hippocampus	7.52 ± 1.67	8.26 ± 3.12	1.236	<0.001	Hippocampus	7.74 ± 2.13	8.93 ± 2.18	0.127	<0.001
TH	16.64 ± 4.98	16.46 ± 5.63	2.223	0.459	TH	16.72 ± 5.62	19.19 ± 5.14	0.982	<0.001
GP	8.16 ± 2.66	8.67 ± 2.11	0.871	0.147	GP	9.63 ± 3.16	9.76 ± 2.15	1.667	0.335
SN	0.96 ± 0.23	0.96 ± 0.15	1.287	0.993	SN	0.97 ± 0.17	0.97 ± 0.23	1.093	0.567
Putamen	13.27 ± 4.56	13.29 ± 5.18	6.334	<0.001	Putamen	12.72 ± 3.89	14.23 ± 3.79	5.761	<0.001
RN	0.66 ± 0.21	0.69 ± 0.24	1.732	0.564	RN	0.73 ± 0.14	0.76 ± 0.15	1.004	0.761
CN	12.29 ± 3.91	12.23 ± 4.91	8.225	0.432	CN	14.82 ± 4.87	15.18 ± 3.21	5.668	<0.001
Whole brain volume	1473.62 ± 127.68	1518.67 ± 97.68	17.623	<0.001	Whole brain volume	1485.46 ± 79.65	1523.38 ± 113.32	12.683	<0.001
**≥10–≤14 years old**					**≥14–≤18 years old**				
Frontal	168.46 ± 34.54	173.25 ± 28.67	5.709	<0.001	Frontal	179.65 ± 51.23	182.68 ± 49.93	3.872	<0.001
Temporal	155.43 ± 36.89	166.19 ± 39.54	6.238	<0.001	Temporal	166.87 ± 38.92	179.11 ± 57.82	21.653	<0.001
Hippocampus	7.87 ± 2.35	8.46 ± 3.11	2.334	<0.001	Hippocampus	8.12 ± 2.13	8.92 ± 3.09	3.885	<0.001
TH	16.59 ± 4.55	17.23 ± 6.23	1.984	<0.001	TH	16.39 ± 5.69	18.56 ± 5.67	7.623	<0.001
GP	8.52 ± 2.89	8.49 ± 2.05	4.327	0.337	GP	8.47 ± 2.35	8.36 ± 2.91	2.138	0.147
SN	0.98 ± 0.23	1.09 ± 0.21	1.006	<0.001	SN	0.96 ± 0.21	1.14 ± 0.45	1.554	<0.001
Putamen	11.34 ± 3.78	12.45 ± 3.23	3.023	<0.001	Putamen	11.38 ± 4.47	12.47 ± 5.12	3.892	<0.001
RN	0.69 ± 0.13	0.74 ± 0.19	3.448	<0.001	RN	0.71 ± 0.18	0.73 ± 0.32	0.983	<0.001
CN	12.39 ± 4.67	13.56 ± 7.21	6.981	<0.001	CN	13.39 ± 5.21	14.78 ± 4.87	4.679	<0.001
Whole brain volume	1540.09 ± 141.53	1652.19 ± 117.65	13.793	<0.001	Whole brain volume	1576.79 ± 127.59	1667.21 ± 157.62	9.894	<0.001

*TH, Thalamus; GP, Globus pallidus; SN, Substantia nigra; RN, Red nucleus; CN, Caudate nucleus*.

**Figure 5:**
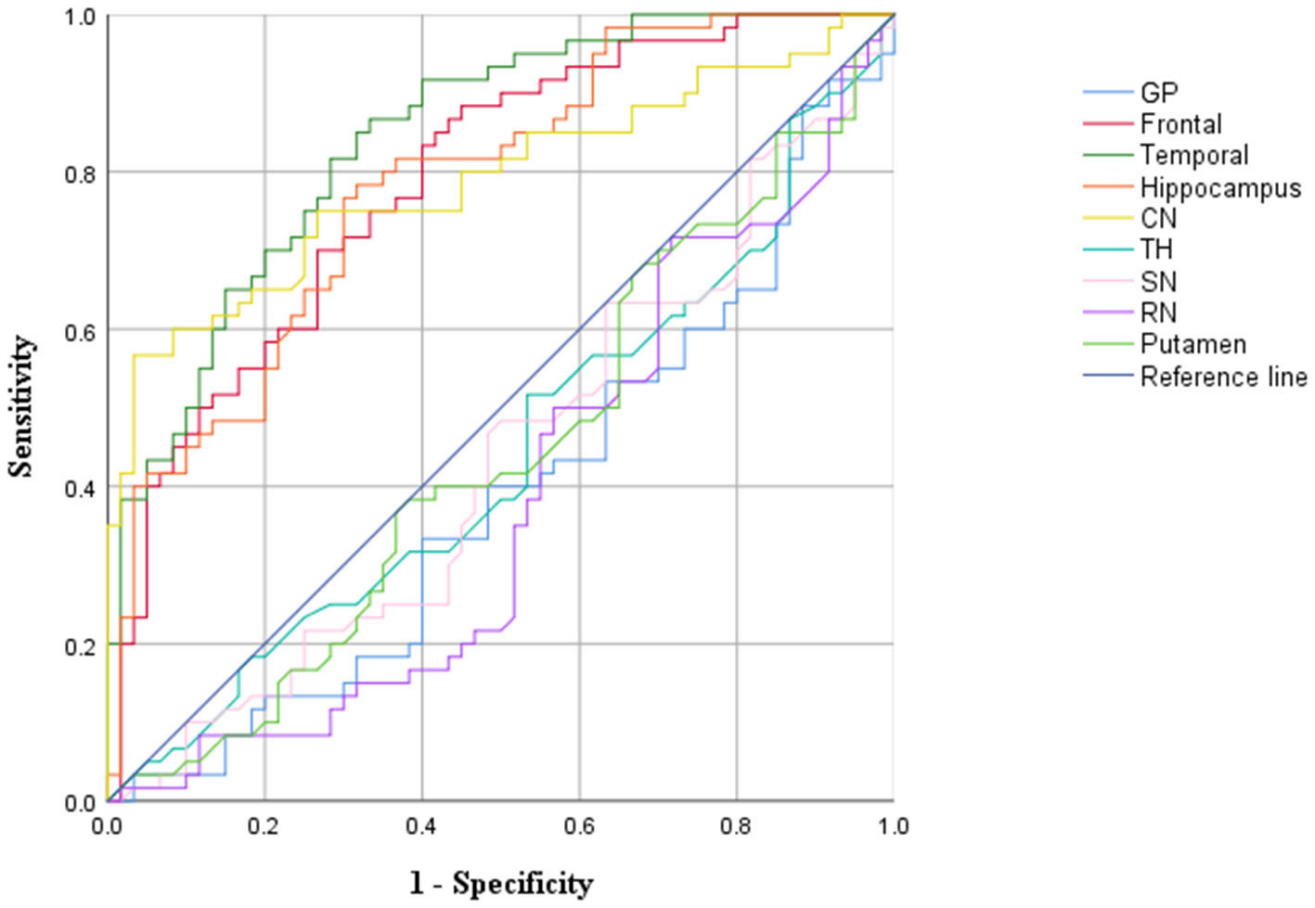
ROC curve analysis results of CBF values in brain region.

The ROC analysis results showed that the CBF values of the frontal lobe, hippocampus, temporal lobe, and caudate nucleus could distinguish children with autism (AUC > 0.05, *P* < 0.05), and the AUC value of the temporal lobe was the highest (Figure 5, Table 4).

**Table 4:** ROC curve analysis results of CBF values in brain regions (*n* = 320).

**Brain region**	**AUC**	**Std. error**	***P*** **-value**	**95% CI**	
				**Lower bound**	**Upper bound**
Frontal	0.787	0.041	0.000	0.707	0.867
Temporal	0.843	0.035	0.000	0.775	0.911
Hippocampus	0.783	0.041	0.000	0.703	0.864
TH	0.440	0.053	0.255	0.337	0.543
GP	0.389	0.051	0.067	0.289	0.490
SN	0.435	0.057	0.218	0.332	0.538
Putamen	0.442	0.053	0.272	0.339	0.545
RN	0.381	0.052	0.075	0.280	0.473
CN	0.789	0.042	0.000	0.339	0.545

*TH, Thalamus; GP, Globus pallidus; SN, Substantia nigra; RN, Red nucleus; CN, Caudate nucleus*.

## Discussion

The study results imply that the decrease in the CBF value in children with autism will increase with age. The decreases in CBF values in these brain regions may be related to cognitive, language, and motor retardation in children with autism (when a child with autism is ~2 years old, compared with a healthy child, the gaps in cognitive, language, and motor development are smaller. As the age of children with autism increases, the gaps between their cognitive, language, and motor development gradually increases compared with that in healthy children, eventually leading to a decrease in blood perfusion and a gradual increase in the number of affected brain regions) (20–23).

The study results show that except for the 2-year-old and 5-year-old groups, the CBF value of the frontal lobe in autistic children in the other groups was lower than that in healthy children, and the CBF value for blood perfusion in the 3-year-old group was significantly lower than that in the 4-year-old group. The reason for this phenomenon may be that the growth and development of the frontal lobe of autistic children begin to slow down from the age of ~3 years, resulting in a decline in frontal lobe blood flow and perfusion, and then the growth and development of the frontal lobe gradually recover and finally return to normal at the age of ~5 years. However, after the age of 6 years, the frontal lobe's blood flow and perfusion of children with autism gradually decrease (24–27).

In this study, we selected the basal ganglia, frontal lobe, temporal lobe, hippocampus, and other brain regions as key research areas because these are important neurological functional areas (28). The basal ganglia of the brain, which is located deep in the brain, controls and regulates motor functions together with the cerebral cortex and cerebellum (29). The frontal lobe is the most developed brain lobe and has a complex structure and extensive neural connections, including movement, memory, judgment, analysis, thinking, and other functions (30). Abnormalities in the frontal lobe can cause abnormalities in movement, memory, and other functions (31). The hippocampus is closely related to the functions of learning, memory, emotion, and movement, and abnormalities in the hippocampus can cause abnormalities in its functions (32, 33). The results of the study showed that the CBF values of the caudate nucleus, basal ganglia, hippocampus, frontal lobe, temporal lobe, and other brain regions were lower than those in healthy children. The decrease in the CBF value may affect the development of nerve cells, leading to abnormal neurodevelopment in the brain and ultimately leading to abnormal behavior in children with autism (34, 35).

The study results show no statistically significant difference in the volume of the same brain region in children with autism at the same age in the 2- to 5-year-old group, however, the CBF value for blood perfusion in some brain regions of children with autism of the same age was lower than that in the corresponding brain regions of healthy children, suggesting that the decrease in the CBF value for cerebral perfusion in some brain regions of children with autism is unrelated to the volumes of brain regions and may be related to cognitive, language, and motor retardation in children with autism (36, 37); the study results show that in the 6- to 18-year-old group, the volumes of some brain regions of healthy children in the same age group were lower than those in the corresponding brain regions of children with autism, possibly because as the ages of children with autism increases, cerebral blood perfusion decreases, the number of affected brain regions increases, and long-term cerebral blood perfusion is insufficient, which eventually leads to abnormal brain regions (38, 39).

The study results show that the temporal lobe, frontal lobe, hippocampus, and caudate nucleus can distinguish children with autism (AUC > 0.5, *P* < 0.05). Therefore, these four brain regions can be used as key brain regions for brain imaging diagnosis in children with autism. If the CBF values of the above four brain regions are reduced and the clinical symptoms are consistent with the characteristics of autism, the clinician can diagnose the child with autism and give reasonable treatment in time to improve the condition of the child.

### Limitations of This Study

This study is not a multicenter study, and the results of the study may be regional; some of the children included in the study were examined after sedation, which may affect the state of cerebral blood flow and ultimately lead to a certain deviation in the study results (Ogawa found that patients' brain CBF values decreased after sedation). The above shortcomings will be further studied in the future (40, 41).

In summary, 3D-PCASL shows lower brain CBF values in children with autism, and with increases in the ages of children with autism, the number of brain regions with decreased CBF values gradually increases.

## Data Availability Statement

Publicly available datasets were analyzed in this study. This data can be found here: Figshare, https://figshare.com/s/9ecf5f9eeafd809fe53b.

## Ethics Statement

The study protocol was approved by the Human Ethics Committee of the Children's Hospital of Chongqing Medical University (No. 2018-47). Written informed consent to participate in this study was provided by the participants' legal guardian/next of kin.

## Author Contributions

ST, ZP, and LH: experimental design and project management. XL: statistical analysis and image analysis. QR and LW: data acquisition and data analysis. LN: software support. All authors contributed to the article and approved the submitted version.

## Funding

This study was supported by the Chongqing Municipal Education Commission (No. KJQN202000425) and National Clinical Research Center for Child Health and Disorders (No. NCRCCHD-2021-YP-07).

## Conflict of Interest

LN was employed by GE Healthcare and MR Research China. The remaining authors declare that the research was conducted in the absence of any commercial or financial relationships that could be construed as a potential conflict of interest.

## Publisher's Note

All claims expressed in this article are solely those of the authors and do not necessarily represent those of their affiliated organizations, or those of the publisher, the editors and the reviewers. Any product that may be evaluated in this article, or claim that may be made by its manufacturer, is not guaranteed or endorsed by the publisher.

## Abbreviations

MRI: magnetic resonance imaging
3D-PCASL: three-dimensional pseudocontinuous arterial spin labeling
ASD: autism spectrum disorder
DSM-V: Diagnostic and Statistical Manual of Mental Disorders, Fifth Edition
MCV: mean corpuscular volume
VBM: voxel-based morphology
CBF: cerebral blood flow
PLD: post labeling delay
FLAIR: fluid attenuated inversion recovery
T2WI: weighted imaging
TR: repetition time
FOV: field of view
TE: time of echo
NEX: number of excitations.
